# The Effects of Acupressure Applied After Bariatric Surgery on Gastrointestinal Functions, Pain, and Anxiety

**DOI:** 10.1007/s11695-025-07768-x

**Published:** 2025-05-22

**Authors:** Duygu Balaban, Ayşegül Yayla

**Affiliations:** 1 Department of Surgical Nursing, Kocaeli Research Hospital, Kocaeli, Turkey; 2 Department of Surgical Nursing, Faculty of Nursing, Atatürk University, Erzurum, Turkey

**Keywords:** Pain, Acupressure, Anxiety, Gastrointestinal functions, Nurse

## Abstract

**Background:**

This study was conducted to determine the effects of acupressure applied to patients after bariatric surgery on gastrointestinal functions, pain, and anxiety.

**Methods:**

The study was conducted as a randomized controlled experimental trial with a placebo group. The research data were collected at the General Surgery Clinic of Private Aktif Kocaeli Hospital between January 2023 and March 2024 from 90 patients (30 in the control group, 30 in the intervention group, and 30 in the placebo group) who underwent bariatric surgery. The “Patient Descriptive Form,” “Postoperative Gastrointestinal Functions Assessment Form,” “Visual Analog Scale,” “Verbal Pain Scale,’’ and “State-Trait Anxiety Inventory” were used in data collection. The data were analyzed using the SPSS 22 package program, and the results were interpreted at a *p* < 0.05 significance level.

**Results:**

Of the patients in the intervention group, 63.3% flatulated at the 12th postoperative hour, 43.3% passed stool, and there was a significant difference between the groups (*p* < 0.05); they consumed more food daily (*p* < 0.05), their pain (3.43 ± 0.97) and distension (3.20 ± 1.06) scores were lower, and more patients (46.7%) experienced mild pain (*p* < 0.05). Although there was no statistically significant difference between the mean nausea scores of the groups at the 6th, 12th, 24th, and 48th postoperative hours, the mean nausea scores of the patients in the intervention group at the 12th postoperative hour (0.33 ± 5.07) were lower than those of the control (1.33 ± 1.83) and placebo groups (1.33 ± 4.34) (*p* > 0.05). All three groups had similar mean state-trait anxiety scores (*p* > 0.05).

**Conclusion:**

The study found that acupressure applied after bariatric surgery ensured that patients flatulated and passed stool in the early period, increased food consumption, and reduced abdominal distension and pain. In line with these results, it can be recommended that acupressure be applied in clinics after surgery.

## Introduction

Obesity is a severe disease that adversely affects people’s health and increases mortality and morbidity rates worldwide [4]. The World Health Organization (WHO) describes obesity as the excessive accumulation of fat causing health risks. Obesity also increases the risk of developing various diseases, such as cancer, diabetes mellitus, and cardiovascular diseases [6, 19].

Medical and surgical treatments are used to treat obesity. Medical treatment includes therapy methods such as drug use, diet, physical activity, and behavioral changes, whereas surgical therapy is performed when adequate/intended weight loss cannot be achieved using these methods [39]. Weight loss surgery, also known as metabolic and bariatric surgery (MBS), is an effective weight loss treatment and is associated with reduced mortality and improvements in obesity-related health conditions and quality of life [5]. Metabolic and bariatric surgery has progressively become safer, along with advancements in laparoscopic technologies. Nevertheless, it still causes potential complications [2]. Thus, gastrointestinal problems, including nausea and vomiting [24, 35], abdominal distension [23], slowing down of bowel movements [16], and delayed oral intake [29, 31], may arise after bariatric surgery, which is a surgical procedure. Postoperative pain is an acute pain that begins with surgical trauma and decreases over time with wound healing. Studies show that approximately 30–80% of patients experience pain at varying levels [34]. These postoperative problems lead to undesired conditions such as fear, anxiety, and insomnia in patients and decrease their quality of life. This prolongs patients’ recovery time and hospital stay. Furthermore, patients’ health expenses increase [1, 13].

The care provided by nurses as members of a multidisciplinary team is essential for the success of metabolic and bariatric surgical procedures [15]. Postoperative unfavorable events should be controlled to maximize recovery after bariatric surgery. To this end, non-pharmacological methods can be employed in addition to pharmacological methods [28]. Music therapy, aromatherapy, reflexology, ozone therapy, acupuncture, and acupressure can be applied as non-pharmacological methods [11].

According to traditional Chinese medicine, acupressure refers to an easily applicable non-pharmacological method without adverse effects that is implemented through tactile pressure using hands, fingers, thumbs, or small beads, balances the energy in the body, and cures diseases [10]. Acupressure, which is classified in nursing interventions, can be applied to prevent nausea, ensure relaxation, and decrease pain [7]. In addition, it is indicated to effectively recover gastrointestinal tract functions [8]. Studies have stated that acupressure increases patients’ comfort during surgical interventions and may reduce the rate of postoperative complications [28]. Qin et al. [27] reported an improvement in bowel movements due to applying acupressure after abdominal surgery. Soylu [30] conducted a study on 132 patients undergoing laparoscopic cholecystectomy and found a further increase in bowel sounds of patients due to the massage applied to acupressure points ST 25, CV 12, and HT7 after surgery. A meta-analysis reported that further experimental studies should investigate the effects of acupressure on intestinal functions [18],Y.-H. [22]. In their study on the impact of acupressure applied to patients after laparoscopic cholecystectomy on pain, Sürücü et al. [33] indicated that acupressure decreased pain. Another study on patients who underwent open-heart surgery found that acupressure reduced pain [32]. In their study investigating the effects of acupressure on anxiety and physiological parameters in six types of elective surgeries, Wang et al. [36] determined that acupressure reduced patients’ cortisol levels. Studies have demonstrated that applying acupressure, a safe and effective method, to different groups of patients after surgery may decrease pain, improve GI motility, and reduce anxiety. The literature review found no study examining the effects of acupressure on gastrointestinal problems, pain, and anxiety after metabolic and bariatric surgery. Hence, further studies with higher evidence are needed to accept the effectiveness of acupressure applied to patients. We believe the present study will contribute to the literature and health practices. This research was conducted to determine the effects of acupressure applied to patients after bariatric surgery on gastrointestinal functions, pain, and anxiety.

## Research Hypotheses

- **H**_**1**_**:** Acupressure applied to points ST25 and CV12 after bariatric surgery increases flatulence.
- **H**_**2**_**:** Acupressure applied to points ST25 and CV12 after bariatric surgery increases stool passing.
- **H**_**3**_**:** Acupressure applied to points ST25 and CV12 after bariatric surgery reduces distension.
- **H**_**4**_**:** Acupressure applied to point LI4 after bariatric surgery reduces nausea.
- **H**_**5**_**:** Acupressure applied to point SP6 after bariatric surgery reduces abdominal pain.
- **H**_**6**_**:** Acupressure applied to point HT7 after bariatric surgery reduces anxiety.

## Method

### Research Design

**Fig. 1:**
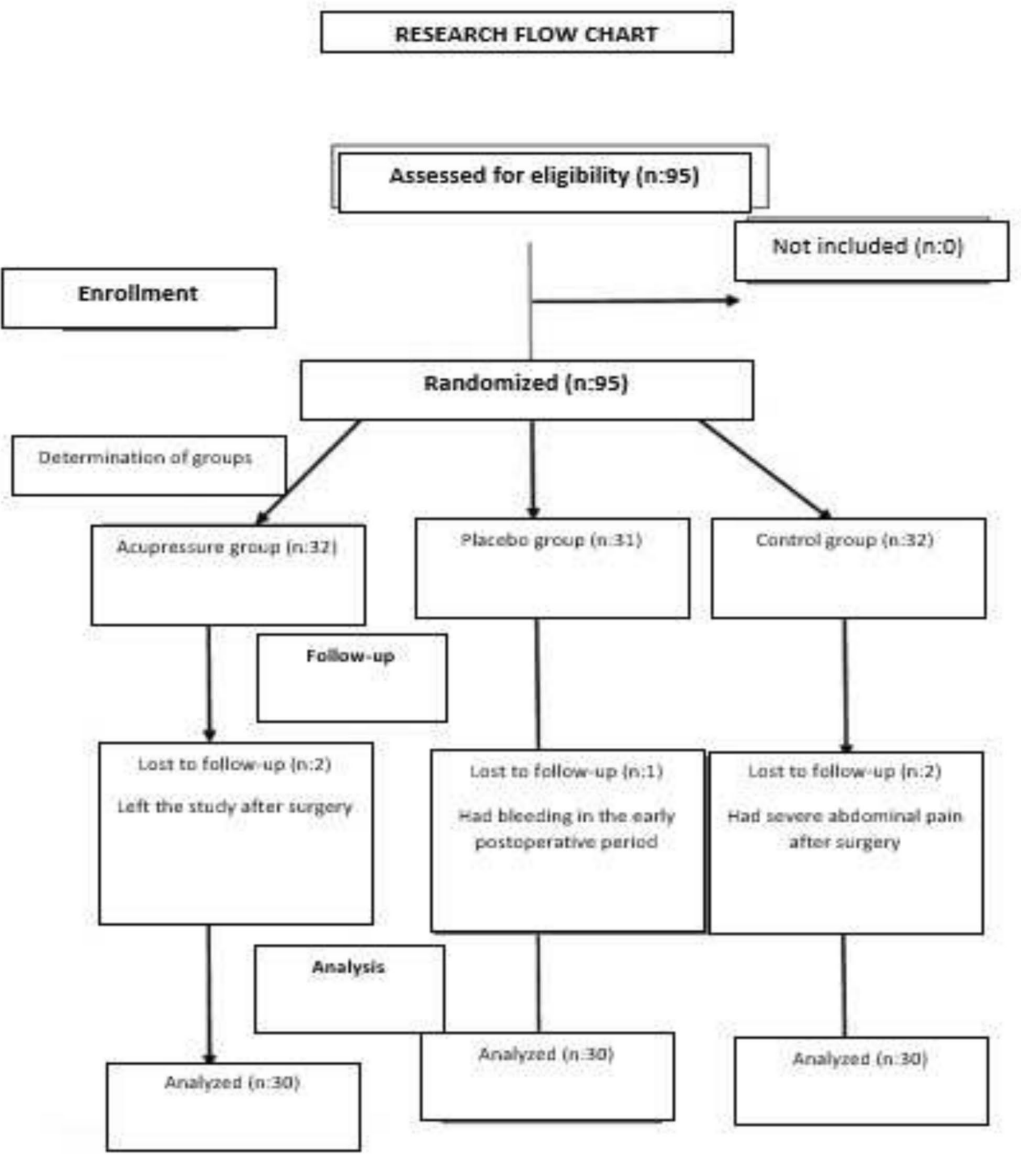
CONSORT flow chart

The research was completed with 90 volunteer patients aged 18–65 who underwent bariatric surgery at the General Surgery Clinic of Private Aktif Kocaeli Hospital between January 2023 and March 2024. Post hoc power analysis was conducted to determine the adequacy of the sample size. The power analysis revealed that the effect size and power of the study were 0.51 and 0.80, respectively, at a significance level of 0.05 and a confidence interval of 95% [9]. We followed the CONSORT statement in the study Fig. 1.

### Participants

The research was conducted as a randomized controlled experimental trial with a placebo group. The study included cooperative patients aged 18–65 who underwent laparoscopic surgery with the sleeve gastrectomy method, had no hearing and speech problems, were open to communication, were conscious, had not previously received a psychiatric diagnosis, and had not previously had constipation problems. Five patients, including one patient with postoperative bleeding, two patients with severe postoperative abdominal pain, and two patients wanting to withdraw from the study, were excluded.

### Acupressure Application

Following the bariatric surgery, patients in the intervention group underwent three sessions of acupressure at the 6 th, 12 th, and 24 th postoperative hours to acupressure points ST25, CV12 (pregnancy meridian), SP6 (Sanyinjiao), HT7 (heart), and LI4 (He Gu). Each acupressure point was massaged for 2 min at 10-s intervals by applying pressure for 20 s. In a session, the application was performed for a total of 16 min, 2 min each, to one point at acupressure point CV12, one point at acupressure point ST25, two points at acupressure points LI4 of both hands, two points at acupressure points HT7 of both hands, and two points at acupressure points SP6 of both feet. Pressure was applied to all acupressure points for a minute to measure patients’ sensitivity and response to pressure prior to the main massage. All points were massaged using the finger cun method. The points were massaged with slow and continuous clockwise movements. Hence, the pressure on the points increased gradually and reached a maximum after 1 min. The finger was kept “at depth” for a few seconds and removed (Bang & Park, 2020). Acupressure was applied to points ST25 and CV12 with rotational movements, while pressure with fingers was applied to acupressure points LI4, SP6, and HT7.

### Data Collection Tools

Twenty-four hours after the surgery, abdominal distension and the severity of nausea were evaluated using the Visual Analog Scale, pain was assessed using the Visual Analog Scale and Verbal Pain Scale, and patients’ anxiety was evaluated using the State-Trait Anxiety Inventory.

### Statistical Analysis

The research data were analyzed using the SPSS for Windows 22 package program. Numbers, percentages, minimum and maximum values, and means and standard deviations were employed in data analysis. The normality distribution of the data was examined with kurtosis and skewness coefficients. When skewness and kurtosis values are between − 1.5 and + 1.5, it is considered that data are normally distributed [25]. In the comparison of pairwise groups, the independent sample *t*-test (normally distributed) and Mann–Whitney *U* test (non-normally distributed) were performed. In the intragroup comparisons of pairwise groups, the dependent sample *t*-test (normally distributed) and Wilcoxon test (non-normally distributed) were conducted. In the comparison of multiple groups, analysis of one-way ANOVA/variance (LSD in normal distributions as advanced analysis, Dunnet’s C in non-normal distributions) and Kruskal–Wallis test (Mann–Whitney *U* test as advanced analysis) were performed for normally distributed and non-normally distributed measurements, respectively. Cronbach’s *α* coefficient was used for internal validity. The groups were compared using Dunnett’s C analysis. The results were interpreted at a *p* < 0.05 significance level.

### Ethical Considerations

The Ethics Committee of Atatürk University Faculty of Medicine (Ethics Committee No: B.30. 2.ATA.0.01.00/141) approved the study protocol. Furthermore, the Clinical Trials registration of the study was done with number “NCT06468345.” Patients were informed about the ethical principles of “Informed Consent” and “Respect for Autonomy,” indicating they were free to participate in the study. Patients were told that their information would be kept confidential based on the ethical principle of “Privacy and Protection of Privacy.” The study adhered to the Declaration of Helsinki on Human Rights since individual rights should be protected in the research.

## Results

The study found that there was no statistically significant difference between the groups in terms of characteristics such as age, sex, marital status, educational status, smoking status, employment status, and the presence of chronic diseases that might impact gastrointestinal functions, anxiety, pain, and distension and that the groups were similar (*p* > 0.05) (Table 1).

**Table 1:** Patients’ descriptive characteristics and their comparison

		Control group		Intervention group		Placebo group		Significance
		*n*	%	*n*	%	*n*	%	
Age	Between 18 and 35	14	46.7	13	43.3	14	46.7	*x*^2^ = 0.090 *p* = 0.956
	Between 36 and 55	16	53.3	17	56.7	16	53.3	
Sex	Female	22	73.3	20	66.7	22	73.3	*x*^2^ = 0.433 *p* = 0.805
	Male	8	26.7	10	33.3	8	26.7	
Marital status	Married	17	56.7	20	66.7	20	66.7	*x*^2^ = 0.861 *p* = 0.650
	Single	13	43.3	10	33.3	10	33.3	
Educational status	Primary school	1	3.3	4	13.3	3	10.0	*x*^2^ = 3.500 *p* = 0.744
	Secondary school	8	26.7	4	13.3	7	23.3	
	High school	10	33.3	12	40.0	11	36.7	
	Undergraduate	11	36.7	10	33.3	9	30.0	
Employment status	Unemployed	17	56.7	16	53.3	19	63.3	*x*^2^ = 0.638 *p* = 0.727
	Employed	13	43.3	4	46.7	11	36.7	
Smoking status	Yes	8	26.7	8	26.7	13	43.3	*x*^2^ = 2.544 *p* = 0.280
	No	22	73.3	22	73.3	17	56.7	
Presence of chronic disease	Yes	1	3.3	3	10.0	-	-	*x*^2^ = 3.663 *p* = 0.160
	No	29	96.7	27	90.0	30	100	

As seen in Table 2, the flatulence of patients in the intervention group at the 12 th postoperative hour was significant. Furthermore, stool passing was significant at the 12 th and 24 th postoperative hours (*p* < 0.05). The oral intake of patients in the intervention group was higher (*p* < 0.05). Patients’ postoperative nausea-vomiting and retching were insignificant (*p* > 0.05).

**Table 2:** Distribution and comparison of gastrointestinal functions at the 6^th^, 12^th^, 24^th^, and 48^th^ postoperative hours

Postoperative			Control group		Intervention group		Placebo group		Significance
			*n*	%	*n*	%	*n*	%	
Flatulence	6^th^ hour	Yes	3	10.0	3	10.0	3	10.0	*x*^2^ = 0.000 *p* = 1.000
		No	27	90.0	27	90.0	27	90.0	
	12^th^ hour	Yes	13	43.3	19	63.3	5	16.7	*x*^2^ = 13.585 *p* < 0.05*
		No	17	56.7	11	36.7	25	83.3	
	24^th^ hour	Yes	22	73.3	26	86.7	18	60.0	*x*^2^ = 5.455 *p* = 0.065
		No	8	26.7	4	13.3	12	40.0	
	48^th^ hour	Yes	29	96.7	26	86.7	27	90.0	*x*^2^ = 1.921 *p* = 0.383
		No	1	3.3	4	13.3	3	10.0	
Stool passing	6^th^ hour	Yes	1	3.3	-	-	1	3.3	*x*^2^ = 1.023 *p* = 0.600
		No	29	96.7	30	100	29	96.7	
	12^th^ hour	Yes	7	23.3	13	43.3	2	6.7	*x*^2^ = 10.949 *p* < 0.05*
		No	23	76.7	17	56.7	28	93.3	
	24^th^ hour	Yes	13	43.3	21	70.0	5	16.7	*x*^2^ = 17.376 *p* < 0.001**
		No	17	56.7	9	30.0	25	83.3	
	48^th^ hour	Yes	19	63.3	23	76.7	22	73.3	*x*^2^ = 1.406 *p* = 0.495
		No	11	36.7	7	23.3	8	26.7	
Vomiting after oral intake	6^th^ hour	Yes	2	6.7	3	10.0	-	-	*x*^2^ = 2.965 *p* = 0.227
		No	28	93.3	27	90.0	30	100	
	12^th^ hour	Yes	-	-	-	-	-	-	*-*
		No	30	100	30	100	30	100	
	24^th^ hour	Yes	1	3.3	-	-	-	-	*x*^2^ = 2.022 *p* = 0.364
		No	29	96.7	30	100	30	100	
	48^th^ hour	Yes	1	3.3	-	-	-	-	*x*^2^ = 2.022 *p* = 0.364
		No	29	96.7	30	100	30	100	
**Nausea after oral intake**	6^th^ hour	Yes	7	23.3	5	16.7	11	36.7	*x*^2^ = 3.271 *p* = 0.195
		No	23	76.7	25	83.3	19	63.3	
	12^th^ hour	Yes	5	16.7	1	3.3	1	3.3	*x*^2^ = 4.957 *p* = 0.084
		No	25	83.3	29	96.7	29	96.7	
	24^th^ hour	Yes	5	16.7	1	3.3	1	3.3	*x*^2^ = 4.957 *p* = 0.084
		No	25	83.3	29	96.7	29	96.7	
	48^th^ hour	Yes	4	13.3	1	3.3	2	6.7	*x*^2^ = 2.169 *p* = 0.338
		No	26	86.7	29	96.7	28	93.3	
Total daily oral intake amount (ml)	24^th^ hour	25 ml	8	26.7	2	6.7	11	36.7	*x*^2^ = 36.618 *p* < 0.001**
		50 ml	11	36.7	5	16.7	7	23.3	
		75 ml	3	10.0	13	43.3	3	10.0	
		100 ml	2	6.7	5	16.7	8	26.7	
		125 ml	-	-	4	13.3	-	-	
		150 ml	6	20.0	1	3.3	1	3.3	
Retching	6^th^ hour	Yes	3	10.0	1	3.3	1	3.3	*x*^2^ = 1.694 *p* = 0.429
		No	27	90.0	29	96.7	29	96.7	
	12^th^ hour	Yes	1	3.3	-	-	-	-	*x*^2^ = 2.022 *p* = 0.364
		No	29	96.7	30	100	30	100	
	24^th^ hour	Yes	1	3.3	-	-	1	3.3	*x*^2^ = 1.023 *p* = 0.600
		No	29	96.7	30	100	29	96.7	
	48^th^ hour	Yes	1	3.3	-	-	-	-	*x*^2^ = 2.022 *p* = 0.364
		No	29	96.7	30	100	30	100	

*p < 0.05; **p < 0.001

Table 3 shows that the difference in mean pain scores, distension, and verbal pain responses at the 24 th postoperative hour between the three groups was statistically significant (*p* < 0.05).

**Table 3:** Comparison of mean pain, distension, and verbal pain scores at the 24 th postoperative hour

	**Control group** (a)			**Intervention group** (b)			**Placebo group** (c)			
	*n*	X ± SD		*n*	X ± SD		*n*	X ± SD		Significance
Pain score	30	6.20 ±	1.54	30	3.43 ±	0.97	30	5.70 ± 1.91		*F* = 28.021 *p* < 0.001** *a*, *c* > *b*
Distension score	30	5.67 ±	1.71	30	3.20 ±	1.06	30	5.40 ± 2.18		*F* = 18.782 *p* < 0.001** *a*, *c* > *b*
				Control group		Intervention group		Placebo group		Significance
				*n*	%	*n*	%	*n*	%	
Verbal pain scale	0 No pain			-	-	-	-	-	-	*x*^2^ = 31.262 *p* < 0.001**
	2 Very mild pain			-	-	-	-	-	-	
	4 Mild pain			-	-	14	46.7	3	10.0	
	6 Severe pain			8	26.7	10	33.3	10	33.3	
	8 Very severe pain			15	50.0	6	20.0	9	30.0	
	10 Unbearable pain			7	23.3	-	-	8	26.7	

*p < 0.05; **p < 0.001

As seen in Tables 4 and 5, no significant results were found with respect to nausea-vomiting and anxiety at the 24 th postoperative hour (*p* < 0.05).

**Table 4:** Comparison of mean postoperative nausea scores by time

Postoperative nausea score	Control group			Intervention group			Placebo group				
	*n*	X ± SD		*n*	X ± SD		*n*	X ± SD			Significance
6 th hour	30	1.46 ±	13.83	30	1.40 ±	15.22	30	2.10 ±	16.47		*F* = 1.935 *p* = 0.151
12 th hour	30	1.33 ±	1.83	30	0.33 ±	5.07	30	1.33 ±	4.34		*x*^2^_KW_ = 1.062 *p* = 0.588
24 th hour	30	0 ±	0	30	0 ±	0	30	0 ±	0		-
48 th hour	30	0 ±	0	30	0 ±	0	30	0 ±	0		-
Significance		*Z* = − 3.773 *p* < 0.001**			*Z* = − 3.870 *p* < 0.001**			*Z* = − 4.051 *p* < 0.001**			

*p < 0.05 **p < 0.001

**Table 5:** Comparison of mean State-Trait Anxiety Inventory scores at the 24 th postoperative hour

	**Control group**		**Intervention group**		**Placebo group**			
	***n***	**X ± SD**	***n***	**X ± SD**	***n***	**X ± SD**		**Significance**
**State anxiety**	30	47.60 ± 8.9	30	45.83 ± 14.1	30	49.77 ± 11.03		*F* = 0.886 *p* = 0.416
**Trait anxiety**	30	30.10 ± 5.92	30	31.03 ± 7.02	30	32.43 ± 9.48		*F* = 0.713 *p* = 0.493

## Discussion and Conclusion

The results obtained from the present study, conducted to reveal the effects of acupressure on gastrointestinal functions, pain, and anxiety after bariatric surgery, were compared with similar research findings and the relevant literature.

The similarity of patients undergoing bariatric surgery with respect to educational status, age, sex, marital status, smoking status, occupation, and the presence of chronic disease demonstrates that the acupressure applications performed in the study are important for effectiveness.

Considering the flatulence rates at the 6 th, 12 th, 24 th, and 48 th postoperative hours, patients in the intervention group flatulated earlier at the 12 th postoperative hour compared to the other groups. Acupressure applied to points CV12 and ST25 after surgery creates pressure on the rectum and relaxes the intestines [37]. Furthermore, acupressure increases bowel movements and reduces the colon transit time of food. Liu et al. [21] study investigating the impacts of acupressure applied to point ST-36 on postoperative GI functions in patients with colorectal cancer and reporting similar results to the present study found significantly more bowel movements in the intervention group compared to the control group. Consequently, it can be said that applications to acupressure points ST-36 and CV-12 may be an effective and applicable method ensuring early gas and stool passing, which are among the gastrointestinal functions. The result of the current study confirms the hypothesis, “H1: Acupressure applied to points ST25 and CV12 after bariatric surgery increases flatulence.”

Furthermore, the rates of stool passing at the 6 th, 12 th, 24 th, and 48 th postoperative hours were higher, and the stool passing time was shorter in the intervention group. Acupressure applied to points ST25 and CV12 accelerates bowel movements and shortens the stool passing time. Soylu’s [30] study on 132 patients undergoing laparoscopic cholecystectomy found that massage applied to acupressure points ST 25, CV 12, and HT7 after surgery increased the bowel sounds of patients in the intervention group more. As a result of the studies yielding similar results to the literature, it can be stated that massage applied to acupressure points CV12 and ST25 accelerates bowel movements and positively impacts early stool passing. This result confirms the research hypothesis, “H2: Acupressure applied to points ST25 and CV12 after bariatric surgery increases stool passing.”

The postoperative mean distension scores of patients in the intervention group were lower because massage applied to acupressure points CV12 and ST25 accelerates bowel movements and relaxes the abdomen. Hence, it causes earlier gas and stool passing. Durmuş İskender and Çalışkan [12] investigated the effects of acupressure applied to patients with total knee arthroplasty on the severity of distension and found that the mean distension scores of the intervention group were statistically lower compared to the control group. In the study by Liu et al. [21] on 112 adult patients undergoing colorectal cancer surgery, acupressure was applied for 5 days after surgery. The study found significantly lower distension scores in the intervention group than in the control group. It can be said that massage applied to acupressure points CV12 and ST25 accelerates bowel movements and relaxes the abdomen. This can be explained by earlier gas and stool passing in patients. The result obtained from the present study confirms the hypothesis, “H3: Acupressure applied to points ST25 and CV12 after bariatric surgery reduces distension.”

The total daily oral intake of patients in the intervention group was higher at the 24 th postoperative hour. Similar to the study by Karaman (2019), this research found that patients in the intervention group flatulated and passed stool earlier and their bowel movements returned to normal earlier compared to the other groups. This may have contributed to the higher oral intake in the intervention group.

In the study, acupressure was applied to point LI4 to reduce nausea and vomiting. Nevertheless, the incidence of nausea-vomiting and retching after oral intake at the 6 th, 12 th, 24 th, and 48 th postoperative hours was similar in all three groups. However, there were fewer patients with nausea-vomiting and retching after oral intake in the intervention group. Gür et al. [17] investigated the effects of acupressure on pain, nausea-vomiting, and retching in patients undergoing thyroidectomy and found that the mean number of nausea and retching was lower in the intervention group at the 6 th and 12 th postoperative hours compared to the control group. However, a similar study by Yilmaz Şahin et al. [38] revealed that the acupressure bracelet attached to point P6 did not significantly reduce nausea and vomiting in the intervention, control, and placebo groups. The result of the current study did not confirm the hypothesis, “H4: Acupressure applied to point LI4 after bariatric surgery reduces nausea.” Nevertheless, it is of clinical significance that the severity of nausea in the intervention group was lower at the 6 th and 12 th postoperative hours than in the control and placebo groups.

In the study, acupressure was applied to point HT7 to reduce anxiety in patients. Despite similar postoperative State-Trait Anxiety Inventory scores in the three groups, the intervention group had lower mean state anxiety scores. Among similar studies, Esmaeili et al. [14] investigated the effects of acupressure applied to point HT7 on anxiety in patients undergoing open-heart surgery. This study found lower state anxiety scores in patients in the intervention group compared to the control group since the study was conducted in a private hospital. The effective communication of nurses working in the private hospital with patients and the detailed information they provided to patients about the process they would experience in the hospital may have contributed to reducing anxiety and obtaining similar scores in all groups. The results obtained from the present study did not confirm the hypothesis, “H6: Acupressure applied to point HT7 after bariatric surgery reduces anxiety.” Nevertheless, it is of clinical significance that the postoperative state anxiety scores of the intervention group were lower than those of the control and placebo groups.

Acupressure at SP6 targeted pain relief, a critical postoperative issue. Consequently, patients in the intervention group had significantly lower VAS and verbal pain scores compared to patients in the other groups. The literature reports that acupressure applied to patients in different surgical fields, such as obstetrics, gynecological surgery, cardiovascular surgery, general surgery, and laparoscopic surgery, reduces pain intensity [3, 38]. Stimulations of acupressure points activate the pain control system. Endogenous opioids such as enkephalin and beta-endorphin (BE), which are known to be involved in this system, and neurotransmitters such as serotonin and noradrenaline are released, which reduces pain. Acupressure applied to point SP6 decreases intra-abdominal pain and contractions and accelerates recovery [26]. This can be explained by the fact that applications to acupressure points reduce pain with analgesic effect by increasing the release of β-endorphin [20]. These results confirm the hypothesis, “H5: Acupressure applied to point SP6 after bariatric surgery reduces abdominal pain.”

The study determined that patients in the intervention, control, and placebo groups had similar descriptive characteristics. The groups were not homogeneously distributed. Acupressure applied to points ST25 and CV12 reduced distension after bariatric surgery and accelerated bowel movements. The study revealed that massage applied to acupressure point LI4 did not reduce nausea and vomiting after bariatric surgery at a desired level and that massage applied to acupressure point SP6 effectively reduced pain after bariatric surgery. According to the study results, acupressure applied to point HT7 alone reduced anxiety after bariatric surgery insufficiently. Furthermore, acupressure, one of the complementary methods, can be employed as a nursing intervention to manage postoperative pain and improve gastrointestinal functions. It can be recommended that studies be conducted in which acupressure is applied to points CV12, HT7, SP6, and ST25 together with LI4 to facilitate flatulence and stool passing, eliminate distension and pain, and reduce nausea and vomiting after bariatric surgery. It can be suggested that the present study should be conducted with a larger sample group on different types of surgeries.

### Limitations in the Research

In the hospital where this research was conducted, the antiemetic and analgesia protocol after bariatric surgery was applied by clinical nurses to all three groups: intervention, placebo, and control groups. Therefore, the findings of the study present the effects of acupressure, in addition to pharmacological treatment, on pain, nausea, and vomiting, and the amount of analgesic and antiemetic drug consumption. This omission may have led patients to notice whether they were in the control, placebo, or acupressure groups. Along with this awareness, the researcher’s touch and communication during the acupressure application may have influenced the gastrointestinal functions, pain, and anxiety findings in this group. Therefore, there is a need for new studies with acupressure control and placebo in this patient group. Since P6 acupoints were not used in the acupressure application in the study, the direct effect of acupressure on PONV symptoms could not be evaluated. The answers given in the research are limited to the information provided by the patients. The research was applied to patients who volunteered to participate in the research on the General Surgery inpatient floor at Private Aktif Kocaeli Hospital. Therefore, the research results can only be generalized to patients with the characteristics of this sample group.

## Data Availability

The data that support the findings of this study are available from the corresponding author upon reasonable request.

## References

[1] Acar K, Acar H, Demir F, et al. Cerrahi sonrası ağrı insidansı ve analjezik kullanım miktarının belirlenmesi. Acıbadem Üniversitesi Sağlık Bilimleri Dergisi. 2016;(2):89–95.

[2] Aderinto N, Olatunji G, Kokori E, et al. Recent advances in bariatric surgery: a narrative review of weight loss procedures. Annals Med Surgery. 2023;85(12):6091–104. 10.1097/MS9.0000000000001472 PMC10718334 38098582

[3] Akgün M. Sezaryen sonrası ağrı üzerine akupresör’ün etkisinin incelenmesi: Randomize, tek körlü, plasebo kontrollü bir çalışma [Investigation of the effect of acupressure on post-cesarean pain: a randomized, single blinded, plasebo controlled study]. Akdeniz Üniversitesi Sağlık Bilimleri Enstitüsü Hemşirelik Anabilim Dalı. 2019.

[4] Berberoğlu Z, Hocaoglu C. Küresel Sağlık Sorunu ‘Obezite’: Güncel Bir Gözden Geçirme [Global health problem ‘obesity’: a current review]. Celal Bayar Üniversitesi Sağlık Bilimleri Enstitüsü Dergisi 2021;8(3):543–552. 10.34087/cbusbed.886473

[5] Bramante C, Wise E, Chaudhry Z. Care of the patient after metabolic and bariatric surgery. Annals Internal Med 2022;175(5). ITC65-ITC80. 10.7326/AITC202205170 35533387

[6] Blüher M. Obesity: Global epidemiology and pathogenesis. Nat Rev Endocrinol. 2019;15(5):288–98. 10.1038/s41574-019-0176-8. 30814686 10.1038/s41574-019-0176-8

[7] Bulechek GM, Butcher HK, Dochterman JM, et al. Hemşirelik girişimleri sınıflaması. Erdemir F., Kav S., Akman Yılmaz A, Çev). İstanbul: Nobel Tıp Kitabevleri. Türkçeleştirilmiş. 2017;1.

[8] Calik KY, Komurcu N. Effects of SP6 acupuncture point stimulation on labor pain and duration of labor. Iran Red Crescent Med J. 2014;16(10):e16461. 10.5812/ircmj.16461 PMC4270652 25558386

[9] Çapık C. İstatistiksel güç analizi ve hemşirelik araştirmalarinda kullanimi: temel bilgiler [Statistical power analysis and its use in nursing studies: basic information]. Anadolu Hemşirelik ve Sağlık Bilimleri Dergisi. 2014;17(4):268–74.

[10] Çayır Y, ve Tanrıverdi EÇ. Kadın sağlığı ve hastalıklarında akupunktur. Dicle Tıp Dergisi. 2022;49(1):256-63.

[11] Celik A, Ural D, Sahin A, et al. Trends in heart failure between 2016 and 2022 in Türkiye (TRends-HF): a nationwide retrospective cohort study of 85 million individuals across entire population of all ages. Lancet Reg Health Eur. 2023;33:100723. 10.1016/j.lanepe.2023.100723. 10.1016/j.lanepe.2023.100723 PMC10636276 37953995

[12] Durmuş İskender M, Çalışkan N. Effect of acupressure and abdominal massage on constipation in patients with total knee arthroplasty: a randomized controlled study. Clin Nurs Res. 2022;31(3):453–62. 10.1177/10547738211033917 34315242

[13] Erden Yüksekkaya S, Akçalı D, Çizmeci P, İnan N, Babacan A. Comparison of the results of a one-day pain questionnaire applied to patients in a university hospital in 2007 and 2012. Gazi Med J. 2015;26(2):52–5.

[14] Esmaeili A, Khoram MR, Gholami M, et al. Pistachio waste management using combined composting-vermicomposting technique: physico-chemical changes and worm growth analysis. J Clean Prod. 2020;242:118523.

[15] Fan M, Hong J, Cheung PN, et al. Knowledge and attitudes towards obesity and bariatric surgery in Chinese nurses. Obes Surg. 2020;30(2):618–29. 10.1007/s11695-019-04173-z. 31758470 10.1007/s11695-019-04173-z

[16] Gagnon L, Sheff E. Outcomes and complications after bariatric surgery. Am J Nurs. 2012;112:26–36. 10.1097/01.NAJ.0000418920.45600.7a. 10.1097/01.NAJ.0000418920.45600.7a 22902899

[17] Gür S, Öztekin SD, Öztekin İ, et al. The effects of Korean hand acupressure on postoperative pain, nausea, vomiting, and retching after thyroidectomy. J Perianesth Nurs. 2025. 10.1016/j.jopan.2024.11.003 40072394

[18] Hsiung W-T, Chang Y-C, Yeh M-L, et al. Acupressure improves the postoperative comfort of gastric cancer patients: a randomised controlled trial. Complement Ther Med. 2015;23(3):339–46. 10.1016/j.ctim.2015.03.010. 26051568 10.1016/j.ctim.2015.03.010

[19] Huang SL, Cheng H, Duffield C, et al. The relationship between patient obesity and nursing workload: an integrative review. J Clin Nurs. 2021;30(13–14):1810–25. 10.1111/jocn.15679. 33529423 10.1111/jocn.15679

[20] Karakus Z, Yangoz ST, Ozer Z. The effect of acupressure in the management of cancer-related pain and anxiety: a systematic review. Hacettepe Univ Fac Nurs J. 2022;9(1):64–73.

[21] Liu Y, Chan CWH, Chow KM, et al. Nurse-delivered acupressure on early postoperative gastrointestinal function in patients undergoing colorectal cancer surgery. Asia Pac J Oncol Nurs. 2023;10(5):100229. 10.1016/j.apjon.2023.100229. 37213809 10.1016/j.apjon.2023.100229 PMC10199207

[22] Liu Y-H, Dong G-T, Ye Y, et al. Effectiveness of acupuncture for early recovery of bowel function in cancer: a systematic review and meta-analysis. Evidence-Based Complementary Alternative Med. 2017;2017:1–15. 10.1155/2017/2504021. 10.1155/2017/2504021 PMC5750515 29422935

[23] Ma I, Madura J. Gastrointestinal complications after bariatric surgery. Gastroenterology and Hepatology. 2015;11:526–35. 27118949 PMC4843041

[24] Monkhouse SJ, Morgan JD, Norton SA. Complications of bariatric surgery: presentation and emergency management-- a review. Ann R Coll Surg Engl. 2009;91(4):280–6. 10.1308/003588409X392072 PMC2749388 19344551

[25] Ömeroğlu E, Büyüköztürk Ş, Aydoğan Y, et al. Okul öncesi sosyal beceri değerlendirme performansının piyasaya sürülmesi: güvenilirlik ve güvenirlik analizleri. 21. Yüzyılda Eğitim ve Toplum. 2014;3(8):37–46.

[26] Oncu Celik H. The effect of acupressure application during labor on labor pain and duration of labor (Master's thesis, Health Sciences Institute). 2016.

[27] Qin S, Bai Y, Lim HY, et al. Randomized, multicenter, open-label study of oxaliplatin plus fluorouracil/leucovorin versus doxorubicin as palliative chemotherapy in patients with advanced hepatocellular carcinoma from Asia. J Clin Oncol. 2013;31(28):3501–08. 10.1200/JCO.2012.44.5643 23980077

[28] Sarıtaş S, Kapıkıran G. Ameliyat Öncesi ve Sonrası Dönemde Akupunktur/Akupresur Uygulamaları [Acupuncture/acupressure applications preoperative and postoperative period]. Turkiye Klinikleri Traditional Complementary Med-Special Topics. 2022;3(2):80–4.

[29] Shikora SA, Kim JJ, Tarnoff ME. Nutrition and gastrointestinal complications of bariatric surgery. Nutr Clin Pract. 2007;22:29–40. 10.1177/011542650702200129 17242452

[30] Soylu D. Laparoskopik kolesistektomi ameliyatı sonrası uygulanan akupresürün gastrointestinal sistem fonksiyonları ve anksiyete üzerine etkisi [The effect of acupressure applied after laparoscopic cholecystectomy on gastrointestinal system functions and anxiety]. Erciyes Üniversitesi Sağlık Bilimleri Enstitüsü Hemsirelik Anabilim Dalı. 2020.

[31] Soylu D, TekinsoyKartın P. The effect on gastrointestinal system functions, pain and anxiety of acupressure applied following laparoscopic cholecystectomy operation: a randomised, placebo-controlled study. Complement Ther Clin Pract. 2021;43:101304. 10.1016/j.ctcp.2021.101304. 33540298 10.1016/j.ctcp.2021.101304

[32] Şen S. Kalp cerrahisi sonrası yapılan akupresür uygulamasının ağrı, anksiyete ve uyku kalitesi üzerine etkisi [The effect of acupressure on pain, anxiety and sleep quality after cardiac surgery] (Doctoral dissertation, Sakarya University (Türkiye)). 2018.

[33] Sürücü HA, Topdemir EA, Duman M, et al. Examining the influence of the COVID-19 pandemic process on nurses and society’s perception of the nursing image. Med Rec. 2023;5(3):648–54.

[34] Tura I, Erden S. Postoperatif Ağrı Kontrolünde Kanıt Temelli Öneriler. Dental and Medical J-Rev. 2022;4(1):34–47.

[35] Varner KL, March AL. Prevention of nausea and vomiting after laparoscopic sleeve gastrectomy: are we doing enough?. AANA J. 2020;88(2). 32234206

[36] Wang B, Liu Y, Xiehe S, et al. Four patterns of canine Wei syndrome treated with Traditional Chinese Medicine. Complement Med Res. 2023;30(2):174–80. 10.1159/000528047 36731444

[37] Yıldırım Dİ. Evaluation of the effects of body and auricular acupuncture combination therapy in obesity control: a retrospective study. Cukurova Medical Journal. 2023;48(3):852–8.

[38] Yilmaz Sahin S, Iyigun E, Can MF. Effect of acupressure application to the P6 acupoint before laparoscopic cholecystectomy on postoperative nausea-vomiting: a randomized controlled clinical study. Int J Nurs Stud. 2018;87:40–8. 10.1016/j.ijnurstu.2018.07.011. 10.1016/j.ijnurstu.2018.07.011 30053681

[39] Yüksel A. Bariatrik cerrahi operasyonu geçiren morbid obez bir hastanın 3 yıl sonraki beslenme durumu: Olgu sunumu [Nutritional status of a morbid obese patient after three years of bariatric surgery: case report]. İzmir Kâtip Çelebi Üniversitesi Sağlık Bilimleri Fakültesi Dergisi. 2016;1(1):39–45.

